# Masticatory ability in older individuals: A qualitative interview study

**DOI:** 10.1111/ger.12515

**Published:** 2020-11-27

**Authors:** Per Elgestad Stjernfeldt, Gerd Faxén‐Irving, Inger Wårdh

**Affiliations:** ^1^ Department of Dental Medicine and Academic Centre for Geriatric Dentistry Karolinska Institutet Stockholm Sweden; ^2^ Folktandvården Stockholms län AB Stockholm Sweden; ^3^ Division of Clinical Geriatrics Department of Neurobiology Care science and Society (NVS) Karolinska Institutet Stockholm Sweden

**Keywords:** diet, masticatory ability, qualitative research

## Abstract

**Aim:**

To explore older individuals’ experienced masticatory ability and the impact of masticatory ability in daily life.

**Material and methods:**

This study applied an open‐ended exploratory approach using inductive reasoning. The design was inspired by the qualitative method grounded theory. The final sample consisted of twelve older participants. Seven were men, and five were women. The interviews were audio‐recorded and transcribed verbatim. The interviewer successively read the transcribed data and analysed the material in cooperation with the authors.

**Results:**

Three categories developed from the data; Deteriorating oral health and functional loss, Eating habits, Prosthetic rehabilitation and function. A core category named Adaptation emerged. Adaptation describes how individuals successfully could adapt to a decreased function and in spite of this develop a positive view of their masticatory ability.

**Discussion:**

The participants described an experience of gradually deteriorating oral function that had affected their masticatory ability. By adapting to this functional degradation, some of the participants overcame the functional deficiencies. Most participants perceived their masticatory ability to be good, even though their ability to process some food types was described as inadequate.

**Conclusion:**

The participants had experienced deteriorating oral health and function throughout life, and they overcame this through adaptation by adjusting their eating habits. Even though prosthetic treatment might be considered successful by the participant, this does not necessarily improve dietary habits. Future research should therefore focus on how dental treatment can be combined with other interventions, such as dietary counselling and physiotherapy to recover physiological function.

## 1 INTRODUCTION

One of the primary goals of dentistry, especially prosthodontic dentistry, is to restore and maintain oral function. Masticatory function is one of the aspects of oral function that the dentist needs to consider.

Recent research has pointed to the relationship between masticatory function and health conditions among older people, such as cognitive function^1^, ^2^ and frailty.^3^, ^4^, ^5^ A significant relationship between edentulism and intake of key nutrients has been proposed.^6^ However, a review published in 2002 could only find weak correlations between masticatory function and deficient dietary intakes.^7^ Although subsequently published studies have indicated the existence of such a relationship,^8^, ^9^ a mapping of systematic reviews found no such evidence for chewing difficulty.^10^ The authors did, however, stress the need for further research. It has also been suggested that masticatory function could affect body weight and although some studies have indicated a possible relationship,^11^ this has not been well established.

It should be noted that these reviews include studies that assess either objective or subjective masticatory function, but as of today there is no established method for assessing either of these in a clinical context. Objective masticatory function is the observable capacity to reduce or mix, a food bolus, while subjective masticatory function is the self‐perceived notion of an individual on their ability to masticate solid food. A recently published systematic review concluded that there is no objective method for measuring masticatory function that has been extensively tested for all the measurement properties validity, reliability, measurement error and responsiveness.^12^ The lack of established methods to assess masticatory function could be a reason why previously published systematic reviews concerning mastication and other health factors have yielded inconclusive and conflicting results.^7^, ^8^, ^9^, ^10^

### 1.1 Masticatory function

Masticatory function is defined as the ability of an individual to masticate solid food. This concept can be further divided into two subdomains. The first subdomain is the objective and quantifiable capacity of an individual to comminute or mix solid food. This objective definition is more precisely known as “masticatory performance,” and it has been measured in experimental conditions in several clinical studies. Generally, masticatory performance is measured through comminute tests, in which a brittle test food, like nuts, is masticated for a number of cycles and then the masticatory performance is quantified by assessing how well the test food has been masticated into smaller particles.^12^ Another frequently used method is to assess how well a test food is mixed into a bolus, the so‐called mixing test. For instance, a two‐coloured wax or chewing gum can be used and masticatory performance then assessed by the degree of colour mixture in the bolus. Other methods to assess masticatory performance have also been developed.^12^ For example, an odour sensor device has been used to measure the amount of odour that is released when an odour developing test food Is masticated.^13^

The other subdomain, known as “masticatory ability,” is defined as the perceived or subjectively assessed masticatory function of an individual. Basically, it is the individual's own opinion of how well he or she is able to masticate solid food. This is usually assessed and quantified using questionnaires,^14^ some of which are specifically designed to assess masticatory ability, while others use a subset of questions from a more generalised questionnaire.

While these two definitions—masticatory performance and masticatory ability—could be described as two different aspects of masticatory function, studies have shown that they in fact correlate weakly or not at all.^15^, ^16^, ^17^ For instance, one study did not show a statistically significant correlation between objective measurements and subjective assessment of masticatory function after prosthetic rehabilitation of post‐canine teeth.^17^ The authors concluded that objective measurement tests are preferable both during treatment planning and when evaluating the effect of prosthodontic treatment.

This indicates that masticatory function is a complex phenomenon and that there might be other aspects of importance that have not yet been identified or given enough consideration. Qualitative analysis might therefore be used to explore masticatory function and masticatory ability in particular, and generate new insights through individual experiences.

The aim of this study was therefore to explore older individuals’ experienced masticatory ability and the impact of masticatory ability in daily life.

## 2 MATERIAL AND METHODS

### 2.1 Statement of Ethics

The study was undertaken following the principles of the Declaration of Helsinki and approved by the Regional Ethical Review Board in Stockholm (protocol 2016/5:2, reference no. 2016/6‐31/5). Participants received oral and written information about the study, and were guaranteed confidentiality. Written consent was obtained from all the participants. The participants could decline further participation at any stage of the study, without negative consequences.

### 2.2 Study design

This study applied an open‐ended exploratory approach using inductive reasoning. It was not based on a pre‐existing theory. Instead, the purpose of the study was to generate a theory about masticatory ability in relation to masticatory function and important key variables. The design was inspired by the qualitative method grounded theory (GT).^18^ The study did not fulfil all requirements for a classical GT. For example, during the recruitment we did not let each participant direct the researcher to next participant by recommendation, but otherwise we mainly worked according to the method, which is described in the following text.

GT is a method that is used to create a hypothesis‐generating theory. The data collection, in our case through individual interviews, begins with an open question. During analysis of the transcribed material, which is performed concurrently with data collection, repeated ideas or concepts become apparent. As new data are added, these concepts, often called codes, are constantly compared and reanalysed. The codes can then be grouped into categories and subcategories that could form the basis for a new emerging theory, often based on an emerging core category.

### 2.3 Participant recruitment and data collection

Participants were recruited from a public dental clinic at the Academic centre for geriatric dentistry in the central part of Stockholm, Sweden. This clinic is focused on treating older persons, both with and without daily living support. First, nine individuals were interviewed. They were selected by colleagues working at the clinic and the selection was guided by the parallel analysis work. Initially, a minimum age of 75 years was set as an inclusion criterion. However, during the course of data collection, the age limit was lowered to 65 years to possibly detect the experiences of such functional aspects. This was necessary since the data analysis suggested that the oldest participants (around 85‐90 years) had become more adapted to functional changes in their masticatory ability.

Participants were selected purposively using “snowball recruitment,” in which the data and results from each interview, not the participants’ recommendation, encourage further recruitment.^19^ Recruitment was ongoing and conducted concurrently with data analysis until saturation or no new relevant information was achieved. As data were being analysed, it became evident that it would be of interest to include additional participants who explicitly stated that they had impaired masticatory ability to see whether we could add useful information to the data. Thus, an additional three participants were included.

Pfeiffer's test,^20^ also known as Short Portable Mental Status Questionnaire, consists of 10 items concerning orientation, information, memory and calculation, and is used as a dementia screening tool, but not as a diagnostic tool. The questionnaire was used in the first six interviews to assess whether the participants could answer the interview questions adequately. In the following data collection procedure, this test was not used, as the interviewer was able to conclude that the participants were cognitively fit to answer and discuss the issue of masticatory ability adequately.

The final sample consisted of twelve elderly participants, the first nine between 82 and 90 years old and the following three between 67 and 73 years old. Seven were men and five were women; see Table 1.

**Table 1:** Description of the informants

Participant	1	2	3	4	5	6	7	8	9	10	11	12
Age	83	89	86	88	87	90	89	73	67	72	69	72
Gender	w	m	w	w	m	m	m	m	m	w	m	m
Removable prosthodontics	N	Y	Y	Y	Y	N	N	N	N	N	N	N

### 2.4 Interviewer

All interviews were conducted by one individual, a dentist with many years of clinical experience. This person was not one of the authors. A senior researcher in the team with a good understanding and experience of qualitative research, provided instruction and training in GT. Deeper insights and skills were gained by the interviewer from the literature on the method.

The interviewer had not met any of the participants beforehand, nor did the interviewer have any information about them except gender and age. The interviewer received consent to contact the participant after the interview for further questions, if necessary.

### 2.5 Interview guide

The qualitative face‐to‐face interviews were supported by an interview guide. The guide included a number of topic areas of masticatory ability, food and dental health (Table 2). In GT, it is unlikely that there will be specific questions, but more areas to explore. The participants were asked open questions and the subsequent discussions meandered in different directions depending on what came to the person's mind when reflecting on their masticatory ability. The participants were asked to speak freely about the topic while the interview guide was looked upon more as a list of relevant questions for the interview aim but with the order of the questions spontaneously influenced by the participants. The interviews were audio‐recorded and then transcribed verbatim.

**Table 2:** Interview guide (translated from Swedish)

Questions about Elderly Individuals’ subjective Chewing Ability (Individuals interviewed will be encouraged to speak freely about their chewing ability, the interview guide will be used by the interviewer as a support/ inspiration when needed.)
Will the chewing become different when ageing? Why?
Do you experience that your chewing ability has changed compared to when you were younger?
If so, can you explain this difference?
Do you remember WHEN you noticed any difference?
Do you bite more carefully? What determines how hard you (dare to) bite?
Are you worried that something will crack if you bite too hard? Do you chew with less force as an elderly?
What is your favourite food? Are you able to eat it?
Is your eating/ choice of what to eat, affected by your chewing ability?
Do you avoid anything because it is too hard to chew? Is there something you wish you were able to eat/that you long for?
Do you prefer/choose some customised option?
If you have problems with chewing, have you developed any tricks or methods to facilitate? If so, which ones?
When you eat a meal, do you drink as you eat, or before, or after the meal?
Do you drink between meals? Why? How often?
Do you drink during nighttime? Eat?
Have you lost any tooth/ teeth throughout life?
When did you lose your tooth/teeth?
Do you know WHY you lost your tooth/ teeth? (Extracted because of caries, periodontitis, root‐filled, fractured, trauma, had prosthodontics on it? Did you have toothache/ pain?)
Do you remember if the chewing was affected when you lost the tooth/ teeth? Did you get used to it? If so, after how long time? Did you miss what was extracted (or lost)?
Have you got prosthodontic replacements throughout life? Fixed (cemented) like crowns or bridges, or removeable prosthesis? Implants? Was the chewing affected?
If removeable dentures, do you remove them while eating?
Have you anytime in life had problems with your jaws or temporomandibular joints? Been treated for this? If so, by who? Specialist? Was your chewing ability affected?
Do you have or have you had any splint? anytime in your life?
How did you experience having a splint? Why did you get one? Did it help if you had any problems? Did you use it as it was planned? Stayed in the drawer?
Do you experience dry mouth?
Has your dentist or your doctor said anything about xerostomia?
Do you have any trick to prevent dry mouth? Have you got any tips? Have you worked out own ways?
Do you think that chewing ability is related to dry mouth?
Do you take any medicines against xerostomia, as for instance pills stimulating saliva? Anything else?
Is there anything else in your health that you can see affects the way you chew/ what you are able to eat?
Do you consider that your life quality is related to your chewing ability?
Can you think of anything else that affects your chewing? Or how it changes throughout life?

### 2.6 Pilot study

A pilot interview was conducted in order to test the interview guide and technical equipment. This informant's responses were used in the final analysis as the interview generated relevant data and the participant met the inclusion criteria. The interview was conducted by the same interviewer who would conduct the consecutive interviews.

### 2.7 Data analysis

The authors successively read the transcribed data and reflected over the material. This was the start of the analysis process that proceeded in close collaboration between the interviewer and the authors. Data were collected until saturation was reached. An inductive perspective was applied in the analysis with the aim of searching for what the participants themselves considered to be important. GT is a systematic constant comparative methodology with simultaneous collection and coding of data in order to both make a strategic sample collection and to identify a core category.^19^ First, a manifest or descriptive analysis was performed in which meaningful units or codes were sorted out according to the study aim and interview guide. During this analytic process, the codes were constantly compared anud sorted into categories and subcategories. New participants were recruited based on the gaps identified during this process.

After the data collection was completed, the researchers identified a “core category” or theory model which explored how the participants experienced masticatory ability and the impact of masticatory ability in daily life. This was the latent part of the analysis.

## 3 RESULTS

The interviews were conducted from June 2016 to June 2017. Only the participant and the interviewer were present during the interview. No transcript was returned to the participants for comments. The interviewer did not need to contact the informants after the interview for clarification of data.

Three categories and a core category emerged from the data; see Table 3. They describe the participants’ perceptions and in some cases illustrated with cited text phrases from the interviews. The proportion of participants who expressed the different perspectives is presented by the interview number in parenthesis.

**Table 3:** Categories and core category

Core category	Adaptation—Adjustment and accommodation to a deteriorating oral health and function.		
Categories	Deteriorating oral health and function	Eating habits	Prosthetic rehabilitation and function
Subcategories	Loss of teeth	Avoidance of certain food types	Improved masticatory ability after treatment
	Weakened dentition	Food preparation	Retained dietary habits after treatment
	Retention of food	Adjusted chewing pattern	Removable dentures and bad retention
	Declined oral sensorimotor regulation	Adjusted social behaviour	

### 3.1 Deteriorating oral health and functional loss

This category describes the participant's perception of their dental health and function in general. When discussing masticatory ability, the participants often talked about their oral health and how it had changed throughout life. The majority of the participants could describe gradually deteriorating oral health and functional loss. One aspect of the deteriorating oral health described by all participants was the gradual loss of teeth, a subject often brought up by the participants when discussing oral health and masticatory ability. For some participants, loss of teeth had led to edentulism.

“The bite changes over time and things changed when my teeth started to crack.”

“…they [the teeth] decayed, one after the other…” (interview 3)

“I can chew like I was young here (points), not here (points).” (interview 3)

Many participants also described how they perceived the function of their dentition after restorative treatment and how the teeth had become functionally weakened as a consequence of the treatment. Old teeth, especially restored teeth, were considered to be functionally weaker than they had been at a young age. As one participant described it:

“….now I have quite a few restorations, crowns and implants, and they, no matter how good they are, are never as good as my own teeth!” (interview 7)

This sense of “weakened dentition” affected the participants’ masticatory habits. Given the condition of their teeth, a majority of the participants adjusted their chewing patterns or choice of food.

Many of the informants described the retention of food and hampered oral clearance while eating:

“….food gets stuck here and there, and you have to remove it after you have eaten…and I experience it while I’m eating also, during chewing…” (interview 7)

“….sometimes food ends up under the denture….that is hell…” (interview 1)

A few participants reported that their mouth felt different in old age than before when eating food and processing the food bolus. This could be interpreted as a description of declining oromotor skills.

“the food does not move around my mouth as well as it used to…” (interview 7)

In summary, when the participants spoke about deteriorating oral health and functional loss, the main reasons for this seemed to be tooth loss, a sense that dentition was weakened, retained food under dentures, hampered oral clearance and declined oral motor skills.

### 3.2 Eating habits

This category describes how the participants developed different habits as a result of their masticatory ability. By habits, we mean both basic masticatory behaviours, such as chewing patterns or choice of food, as well as social behaviours, such as avoiding eating in public. All these different habits resulted from the participants adjusting to their masticatory ability.

A common masticatory habit that the participants described was how they avoided certain types of food because of a reduced masticatory ability.

The participants described certain types of food that they no longer could eat or believed they could not eat, and therefore avoided:

“No, I can’t chew apple, for example.” (interview 3)

“I don’t need molars, I can chew everything I want, but I don’t eat carrots.” (interview 11)

Some adjusted by preparing food in different ways, which allowed them to masticate it more easily:

“I make sure the food is eatable; I cut it into finer pieces.” (interview 3)

“ We managed to find a grater that we could grate the carrots with.” (interview 1)

“I can have a biscuit, crush it in my mouth and then swallow it down with coffee.” (interview 4)

Many participants described how they had changed their chewing patterns or the way they chewed in order to masticate the food. Often this would occur because the participants felt that their restored teeth were too weakened to be able to withstand the mechanical forces of chewing, and they therefore needed to adjust their chewing because of this:

“Then I might eat on my right side because I have an artificial tooth on my left side. I don’t know what it’s called…. Yes (a crown) …and then I realize that it might not…It wouldn’t be smart to chew sticky food on that side.” (interview 9)

Some of the participants described the need to be careful when chewing with “frail” restored teeth, since they perceived them to be functionally weakened and more fragile compared to when they were younger.

One participant described: “I’m probably a little more careful… Yes… Since I’m reminded it's there (fixed crown), it's a signal that I need to be careful. For example…if it's….if it's cabbage. I have trouble eating that.” (interview 1).

“don’t dare to chew because I think…it could happen…that they…that they are fixed, but sometimes they feel loose, not that they are falling out, but maybe they [the teeth] are becoming looser….” (interview 3)

One participant described how they adjusted their chewing pattern when eating certain foods they no longer were able to chew with their teeth, and instead used other oral anatomical features (4):

“I can’t crush things. I have to move my tongue against the roof of my mouth and through this gap in my bottom teeth.” (interview 4)

A few participants had also changed their social habits when eating. A reduced masticatory ability may constitute a social stigma when eating and some participants therefore avoided eating with other people or in public spaces. Denture wearers often described a fear of having their removable denture falling out during meals:

“I’ve had horrible experiences when my old denture suddenly came loose in my mouth and I’m sitting there with a big piece of food and the loose denture in my mouth…I was at a restaurant when it happened ……so I had to go to the bathroom and try to fix it…” (interview 1)

In summary, the participants described that they had developed different eating habits to adjust to their masticatory ability by avoiding certain food, by preparing the food in different ways, and/or by adjusting their chewing pattern or social habits.

Many participants also described how retained food or food getting stuck under prosthetic replacements negatively affected their eating habits. This sensation also corresponds to the category of impaired human dignity, which negatively affects adaptation and masticatory ability. The social stigma of losing a removable denture during meals with other people clearly had an impact on these participants’ eating habits. One participant only ate with close relatives while another avoided eating in public.

### 3.3 Prosthetic rehabilitation and function

In this category, the participants described any type of prosthodontic rehabilitation and how it affected their masticatory ability. Participants who had received fixed prosthodontics were generally positive towards the results. One informant described the feeling of eating crispbread for the first time after prosthetic rehabilitation:

“I remember I told myself: Now I’m going home to eat a crispy sandwich with cheese on it. It was a wonderful feeling.” (interview 2)

“…to hear the sound in my mouth….and the taste. It was a fantastic feeling!” (interview 2)

However, a few of the participants described that prosthodontic replacements were never as good as original teeth.

“I have quite a lot of filled teeth, crowns and implants, and even though they are good, they are not as good as my own teeth!” (Interview 7)

The participants who still had difficulties after prosthetic rehabilitation were those using removable dentures. All the participants who described this phenomenon used removable dentures with bad retention. Interestingly, some participants reported that the prosthetic rehabilitation had improved their masticatory ability, but when asked about dietary habits, they reported that they still retained their old habits and had not started to eat previously uneatable food.

Interviewer: Do you avoid any type of food after the new prosthesis?

Participant: No I don't

(Further along in the interview)

Interviewer: Do you eat hard, solid food like carrots or crispy bread?

Participant: No, I don't eat carrots. I don't eat tough food. (interview 11)

In summary, some participants described how prosthodontic rehabilitation had improved their masticatory ability, yet this did not necessarily mean that they had changed their dietary habits. Informants who used removable dentures described that their main difficulty was bad retention.

### 3.4 Adaptation

As the interviews indicate, all the participants had, to one degree or another, experienced gradually deteriorating oral health and functional loss. This deteriorating condition forced the informants to develop new masticatory habits to adapt to this decreased function. After analysing the interviews, it became apparent that the participants had described an ongoing process of adaptation, and this adaptation process would determine how the participants perceived their masticatory ability. An informant who had successfully adapted to reduced function would develop a positive view of their masticatory ability, and vice versa. Several different aspects of adaptation emerged during analysis.

A majority of the participants adapted to a compromised oral function through an active adjustment of habits. This could, for example, be choice of food or how a meal was prepared. Interestingly, a majority of the participants had not really considered that they had actually altered their lifestyle to adapt to reduced masticatory ability. It was during the interview that they became aware that they had made some sort of change, such as adjusting their choice of food. For instance, they stated that they had no problem chewing every type of food but when asked if they avoided any type of food, they answered that they avoid certain hard foods like carrots, or prepare them in a way to make them more chewable. Therefore, some participants would perceive their masticatory ability to be good, but then realise that they had actually dismissed certain types of food since they could not masticate them.

“I can eat everything but I boil my vegetables.” (interview 2)

This phenomenon appeared several times during the interviews when discussing masticatory ability The participants first state to have a normal, unhindered masticatory ability, but then, when asked further questions, also describe how they have adjusted their habits because of their masticatory ability. The participants did not seem to perceive any sorts of problems, even though they took active steps to compensate for the functional loss, which would indicate that they had adapted to this new condition through passive adjustment. This was possible because the progressive loss of function was a process spanning several years or decades of a participant's life and therefore the change did not become drastically apparent. Since the change was not drastically apparent, it did not require any active adjustment to cope with the functional loss.

“…you got used to it eventually….I didn’t experience it as a collapse, but rather it happened slowly, slowly.” (interview 1)

Finally, a few participants were aware of their declined masticatory ability but they accepted it “as part of life” or were, “when looking at life as a whole,” untroubled by it.

“It’s the teeth that are broken, not me.” (interview 11)

“I eat what I buy……And I can adjust.” (interview 11)

“I don’t complain to other people. I’m not especially fragile, but I get angry sometimes. But I have reconciled with life! Eighty‐eight is pretty old! (interview 3)

The three participants who explicitly stated to have impaired masticatory ability had one thing in common. They had experienced a sudden and drastic change in masticatory ability, often because of trauma or dental treatment that involved extraction of multiple teeth due to prosthodontic failure or periodontal problems. A few participants could describe a similar situation. Since the change is sudden and drastic in these cases, these participants described a situation in which they had great trouble adapting.

“I lost everything except the implants.” (interview 12)

“My chewing became worse NOW, most recently, since Christmas! And I’ve used a denture for a long time, since the seventies.” (interview 3)

Another participant described how everything changed after losing multiple teeth following a fall while walking down the street. Interestingly, although this had happened at a young age, she still had difficulty adapting and considered her masticatory ability to be poor:

“…after that I’ve had a real hard time. With my teeth!” (interview3)

## 4 DISCUSSION

**Figure 1:**
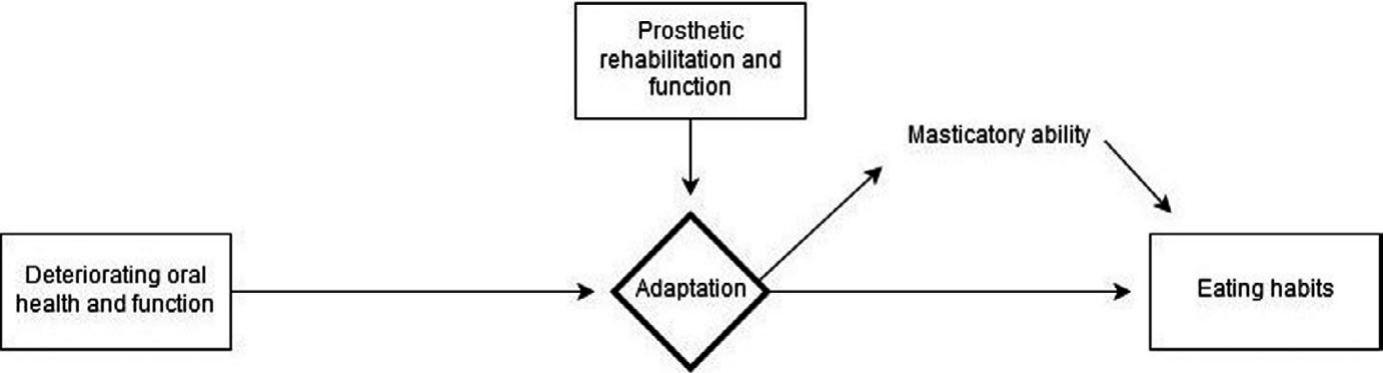
A model of adaptation

At the beginning of this study, we had anticipated to identify specific factors that determined the masticatory ability of the participants. Instead, a flow chart‐inspired theoretical model emerged, which was influenced by three categories (see Figure 1).

The participants described an experience of gradually deteriorating oral health and function, which had affected their masticatory ability. By adapting to this functional degradation, most of the participants had overcome these functional deficiencies and developed a positive view of their masticatory ability. The interviews indicate that most participants perceived their masticatory ability to be good, even though their ability to process some food types was described as clearly inadequate, which resulted in the avoidance of some types of food. It needs to be stressed that *masticatory ability* only describes the participant's own perceived view of their ability to eat solid food.

The term adaptation can be described as an adjustment and accommodation to alteration of function through the lapse of time. The participants in this study described how their masticatory ability had, to one degree or another, gradually deteriorated throughout life. The participants also described how they had adapted to this decreased masticatory ability through different strategies, unconsciously or consciously. Interestingly, a majority of the participants had not really considered that they had actually altered their lifestyle to adapt to a decreased masticatory ability. It was during the interview that they became aware that they had, for instance, adjusted their food choices. Some participants would first perceive their masticatory ability to be good, but then realise that they had actually dismissed certain types of food since they could not masticate them. This is not surprising since the process of gradually deteriorating oral health and functional loss is in many cases a slow process that encompasses an entire lifespan. Most individuals are therefore able to adapt to this loss of function in their daily lives without realising they are taking active steps to adjust. This has been shown in quantitative studies in which individuals with poor masticatory performance actually overestimate their masticatory ability.^21^

When a sudden and unexpected change of function through a catastrophic loss of teeth was experienced, the participants did not report the same ability to progressively adapt^22^ to the loss, and therefore, their masticatory ability was regarded as negative. This is especially important to consider in cases with geriatric patients where multiple extractions are indicated, since the patient may have problems adapting to this drastic change in function following treatment.

From a physiological perspective, it is interesting to note the importance of periodontal mechanoreceptors in providing important proprioceptive information during orofacial motor functions, like chewing.^23^, ^24^ Afferent signalling from these mechanoreceptors plays an important part in the fine motor control of the jaws when manipulating food bolus during mastication. However, these sensory functions are lost with the periodontium when teeth are extracted. It could be theorised that an individual who has lost many teeth would lose this important sensory information from the mechanoreceptors which perturbs oral fine motor control during intraoral manipulation of food^25^ and thus experience a declined masticatory ability. This in turn would adversely affect the ability to adapt, especially if several teeth have been lost within a short period of time.

The process of adaptation affected how the participants perceived their masticatory ability. This self‐perceived notion, either positive or negative, would then affect the participants’ eating habits. Informants who perceived their masticatory ability in a positive manner still adjusted their masticatory habits and adapted by dismissing food that was difficult to chew such as meat, crispy bread and apples or boiled vegetables. An interview study conducted in a multi‐ethnic population‐based sample of adults in the rural United States showed that the participants with severe tooth loss had the lowest dietary quality and avoided certain types of foods.^26^ This suggests that impaired oral health and masticatory ability affect nutritional intake. However, the incitement to adapt a chewing pattern to masticate a food bolus is also affected by other factors, like the fear of breakage of tooth fillings or prosthetically restored teeth.

An interesting finding from this study is that even if a prosthetic treatment was described as a success that had improved the ability to chew food, the eating habits did not seem to have been changed. So even though a prosthetic treatment could be considered a success from both the clinician and patient's point of view, this will not for sure improve the dietary pattern. Some studies have shown that prosthetic rehabilitation can improve masticatory performance; however, this does not necessarily correlate with an improved masticatory ability.^17^, ^27^ It therefore seems, as other authors have suggested, that adjustment to improved dietary habits warrants not only prosthodontic treatment but also multi‐professional collaborations, for instance with dieticians.^28^, ^29^ A cross sectional analysis of UK National Diet and Nutrition Survey 2008‐2014 examined how dental status impacted perceived ability to eat food, nutritional intake and health status.^30^ The authors concluded that it was necessary to focus on developing dental interventions coupled with dietary counselling to encourage the adoption of healthy eating habits in high‐risk population groups. This would indicate that dental interventions should be combined with multi‐professional collaborations, since dental interventions alone may not be enough for success.

General health aspects and medical conditions were not brought up by the participants. None of the participants reflected on the concept of saliva and its possible relationship to their masticatory ability even if many of them reported that they took several medications that could negatively affect their saliva secretion. This could possibly be explained by a normalisation process in which the participants cope with a progressive deterioration of saliva secretion as long as the process is gradual. Since saliva secretion is stimulated during mastication, low saliva secretion might not be perceived as a problem by the participants during the masticatory process, but only during sleep when there is no such stimulation.

In this study, the inclusion criteria of ≤65 years were used as a cut‐off point for “old age.” Yet, there is no general agreement at which age a person becomes old. In Sweden, where this study was conducted, old age is usually considered to start at 65, which is when a person is eligible to receive full economic retirement benefits. However, biological and social ageing present considerable variations at the individual level. The study was performed in a Swedish, urban context and possibly report views from individuals with values that relate to that context. Those values can differ from what older people in different contexts and with other backgrounds experience.

The interviews were performed by a dentist with extensive experience of treating older dental patients and who was not an author of this study. We considered this important, both to be able to ask relevant questions but also to be looked upon as a credible person by the participants. We thought the interviewer would be more objective in the approach if not involved in the design of the research process.

The quality criteria for qualitative research are credibility, transferability, dependability and confirmability.^31^ The term trustworthiness asks the question of whether “the findings can be trusted”.^31^ To ensure credibility, the following strategies were used:

Prolonged engagement: With the help of the interview guide, open questions concerning masticatory ability and other related topics were asked. Follow‐up questions were also used. The raw, transcribed data were analysed continuously until a theoretical model emerged.

Investigator triangulation: All three authors were involved in the methodological design and data analysis process. A consensus on the interpretation of the data was achieved through regular team meetings with all three researchers. Codes, categories and theoretical saturation were also discussed.

Persistent observation: The data were reanalysed several times through a process in which labels and categories were relabelled or revised until an underlying pattern became visible.

The transferability of the study can only be assessed by its readers, and only if the research process is described well enough so that the reader can make an assumption of its transferability to other contexts. The authors have tried to document the study in each step to ensure confirmability. The participants were allowed to speak freely about the subject and it can therefore be difficult tom replicate the results since the information provided are subjective perceptions.

## 5 CONCLUSION

The participants described how they had experienced deteriorating oral health and function throughout life, and they overcame this through adaptation by adjusting their eating habits. This adaption process was often performed unconsciously, and therefore, the participants tended to overestimate their masticatory ability at first. However, during the interview they realised that they had made extensive adjustments to cope with degraded oral function.

Adaptation is affected by several factors, including prosthetic treatment that ultimately affects eating habits, including choice of dietary intake. However, even though prosthetic treatment might be considered successful by the participant, this does not necessarily improve dietary habits. Future research should therefore focus on an intersectoral approach and how dental treatment can be combined with other interventions, such as dietary counselling to improve dietary habits and physiotherapy to recover physiological function.

## CONFLICT OF INTEREST

The authors have no conflicts of interest to declare.

## AUTHOR CONTRIBUTIONS

Per Elgestad Stjernfeldt, Gerd‐Faxén Irving and Inger Wårdh all contributed substantially to this manuscript. Per Elgestad Stjernfeldt and Inger Wårdh conceptualised the study. All authors took part in the analysis and interpretation of the data. All three also contributed to the manuscript drafting and critically revised it for intellectual content.
